# An Integrated Biological Model for Precision Pulmonary Rehabilitation in COPD

**DOI:** 10.3390/arm94050072

**Published:** 2026-10-02

**Authors:** Patrizia Russo, Laura Vitiello, Carlo Tomino, Giorgio Felzani, Massimo Fini, Stefano Bonassi

**Affiliations:** 1 Department of Human Sciences and Promotion of the Quality of Life, San Raffaele University, Via di Val Cannuta 247, 00166 Rome, Italy; patrizia.russo@uniroma5.it (P.R.); stefano.bonassi@sanraffaele.it (S.B.); 2 San Raffaele Sulmona, Viale dell’Agricoltura, 67039 Sulmona, Italy; carlo.tomino@sanraffaele.it (C.T.); giorgio.felzani@sanraffaele.it (G.F.); 3 Clinical and Molecular Epidemiology, IRCCS San Raffaele Roma, Via di Val Cannuta 247, 00166 Rome, Italy; 4 Scientific Direction, IRCCS San Raffaele, Via di Val Cannuta 247, 00166 Rome, Italy; massimo.fini@sanraffaele.it

**Keywords:** chronic obstructive pulmonary disease, pulmonary rehabilitation, precision pulmonary rehabilitation, personalized rehabilitation, systems biology, biomarkers, multi-omics, rehabilitation endotypes, patient stratification, precision medicine

## Abstract

**Highlights:**

**What are the main findings?**

- Pulmonary rehabilitation responsiveness reflects the interaction of genomic, immune, metabolic, behavioural, and environmental factors rather than a single biological mechanism.
- Integrating genomic instability, host genetics, immune competence, behavioural resilience, lifestyle, smoking-related biology, cognition, persistent viral infection, and obesity provides a more comprehensive interpretation of rehabilitation heterogeneity in COPD.

**What are the implications of the main findings?**

- An integrated biological approach may improve patient stratification and support more individualized pulmonary rehabilitation strategies.
- The integration of multi-omics technologies, digital phenotyping, and artificial intelligence may further refine biological profiling and advance precision pulmonary rehabilitation.

**Abstract:**

Pulmonary rehabilitation improves symptoms, exercise capacity and quality of life in chronic obstructive pulmonary disease (COPD), although patients with similar clinical characteristics often show different responses. This review summarizes the biological determinants of rehabilitation responsiveness identified through more than 10 years of translational research. Evidence from longitudinal studies of elderly patients with severe COPD undergoing standardized pulmonary rehabilitation was integrated with clinical, functional, molecular, immunological, cognitive, behavioural and lifestyle data, including long-term follow-up. Baseline DNA damage was markedly increased and further rose during rehabilitation (19.6 ± 7.3% vs. 21.8 ± 7.2% tail intensity) despite significant functional improvement. FKBP5 rs4713916 was the only genetic variant associated with better rehabilitation outcomes. Daily vegetable intake independently predicted treatment success, whereas smoking was associated with oxidative DNA damage, immune alterations and higher Torque Teno Virus (TTV) load. TTV ≥ 4 log10 copies/mL was associated with increased regulatory T cells, reduced α7 nicotinic acetylcholine receptor expression (31% vs. 52%; *p* = 0.023) and shorter 5-year survival (609 ± 160 vs. 937 ± 94 days; *p* = 0.017). Active Coping, Planning and Self-Distraction were the behavioural strategies most strongly associated with rehabilitation response. Cognitive impairment and obesity-related immune regulation further contributed to patient heterogeneity. These observations suggest that rehabilitation responsiveness reflects the interaction of genomic, immune, behavioural and lifestyle factors. Their integration may improve patient stratification and support the development of precision pulmonary rehabilitation.

## 1. Introduction

**Figure 1:**
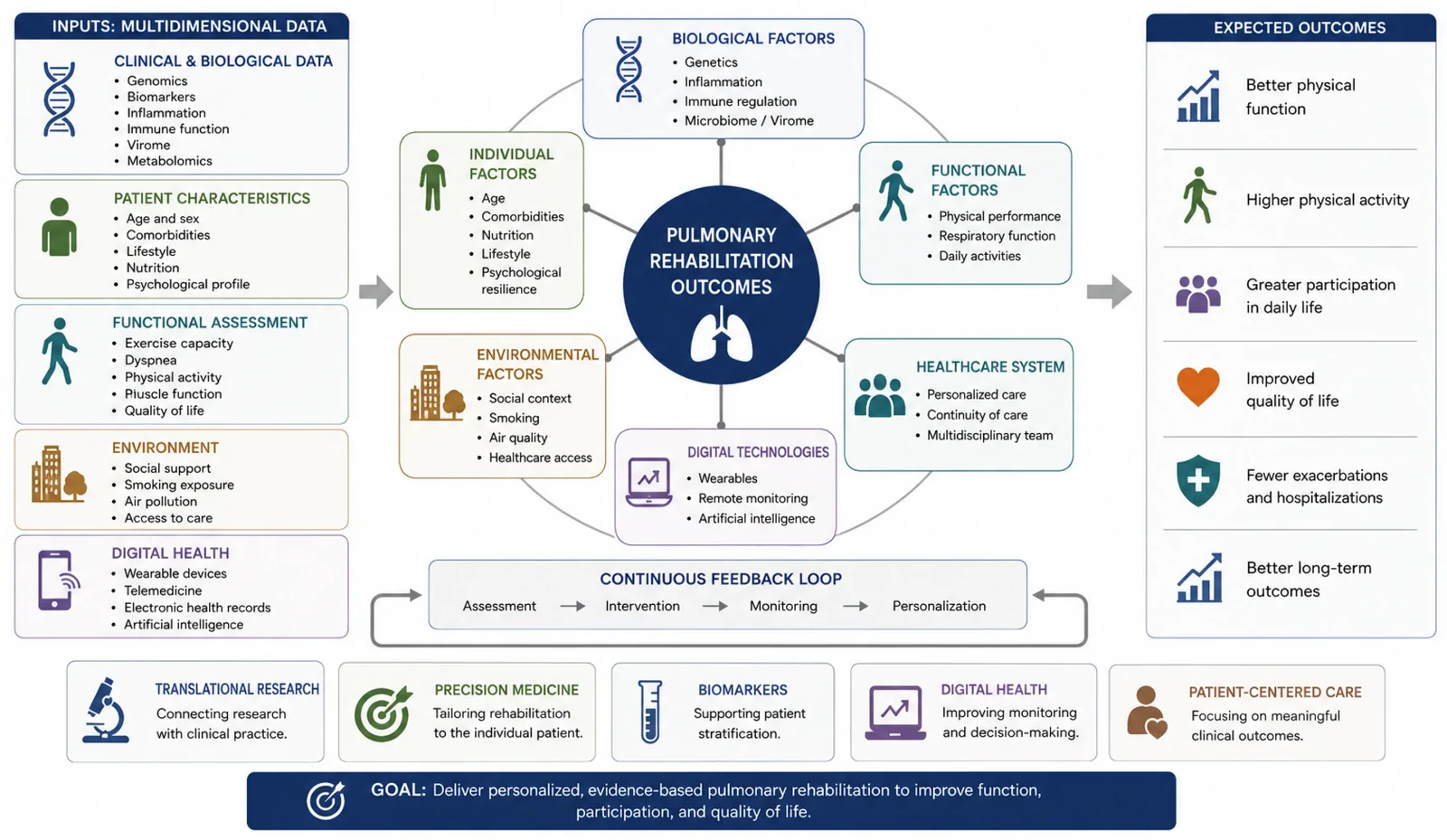
Conceptual evolution toward precision rehabilitation according to the National Institutes of Health (NIH) Research Plan on Rehabilitation [3].

Population ageing and the growing burden of chronic diseases have expanded the role of rehabilitation beyond the recovery of lost function. Rehabilitation is now recognized as an integral component of chronic disease management, aiming to preserve functional capacity, reduce disability, and improve quality of life [1]. This shift is reflected in two major international initiatives. The “World Health Organization (WHO) Rehabilitation 2030: A Call for Action” identifies rehabilitation as an essential element of universal health coverage, [2], while the National Institutes of Health (NIH) Research Plan on Rehabilitation [3] highlights precision medicine, biomarkers, translational research, digital technologies, and interdisciplinary collaboration as priorities for future rehabilitation research. Both initiatives recognize that rehabilitation outcomes result from the interaction of clinical, biological, behavioural, environmental, and social determinants rather than from disease severity alone (Figure 1).

Chronic obstructive pulmonary disease (COPD) exemplifies this complexity. Although persistent airflow limitation remains its defining clinical feature, COPD is increasingly recognized as a multisystem disorder characterized by chronic inflammation, oxidative stress, immune dysregulation, accelerated biological ageing, skeletal muscle dysfunction, nutritional abnormalities, cognitive impairment, psychological distress, and multiple comorbidities. The relative contribution of these processes differs substantially among patients, contributing to the marked heterogeneity observed in disease progression and treatment response [4,5,6,7,8,9,10,11,12,13,14,15].

Pulmonary rehabilitation is one of the most effective non-pharmacological interventions for COPD and consistently improves exercise capacity, dyspnoea, and health-related quality of life [16,17,18,19,20]. Nevertheless, clinically comparable patients frequently achieve different outcomes after rehabilitation. Conventional clinical and functional variables explain only part of this variability [21], suggesting that additional biological mechanisms influence rehabilitation responsiveness.

**Figure 2:**
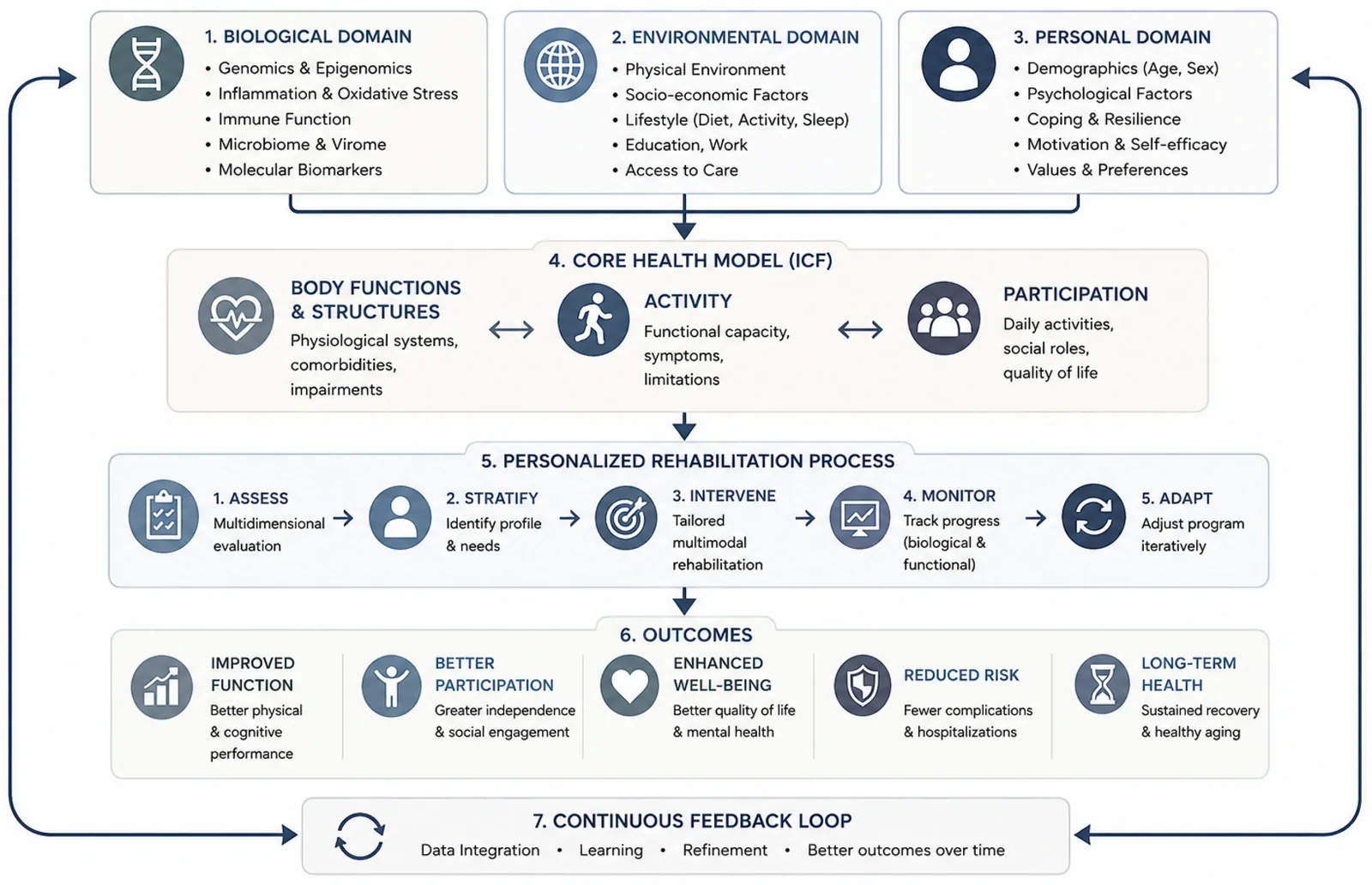
Conceptual model of rehabilomics within the International Classification of Functioning, Disability and Health (ICF).

The concept of rehabilomics, introduced by Wagner and Kumar, proposed the integration of molecular biomarkers into rehabilitation research to better characterize individual variability in functional recovery. Within the framework of the International Classification of Functioning, Disability and Health (ICF), biological markers complement conventional clinical assessment by improving patient stratification and supporting more individualized rehabilitation strategies (Figure 2) [22,23].

Despite this conceptual framework, the application of rehabilomics to COPD remains limited. Most studies have focused on individual biomarkers or single molecular pathways, whereas few have examined how multiple biological processes interact to influence rehabilitation outcomes.

Recent evidence has also highlighted the potential role of biomarkers in exercise-based pulmonary rehabilitation. In particular, inflammatory and oxidative stress markers, as well as emerging markers related to DNA damage, microbiota, and metabolism, may provide additional information on the biological response to rehabilitation and may help identify different patterns of responsiveness [24,25]. However, the available evidence remains limited and heterogeneous, and the relationship between biomarker changes and clinical outcomes is not yet sufficiently established to support their routine use in clinical practice [26].

The studies reviewed here derive from a longitudinal translational research programme initiated in 2013 in elderly patients with severe COPD undergoing standardized pulmonary rehabilitation [7]. The programme progressively expanded to investigate genomic instability, pharmacogenomics, immune competence, chronic viral infection, behavioural resilience, cognition, nutrition, smoking-related biology, obesity, and biological ageing. More recently, transcriptomic, metabolomic, and lipidomic analyses have been introduced to further extend the biological characterization of the cohort.

Although each study addressed a different research question, the findings gradually converged toward a common biological interpretation. Rehabilitation responsiveness could not be explained by any single clinical or biological determinant but appeared to reflect the interaction of multiple biological processes acting at different levels of disease complexity.

This article brings together the findings generated by our longitudinal translational research programme and places them within the broader context of precision pulmonary rehabilitation, rehabilomics, systems biology, and personalized medicine. Rather than providing a systematic review of the literature, the aim is to integrate complementary observations from our research programme into a biological framework that may help explain heterogeneity in rehabilitation responsiveness and generate hypotheses for future validation.

## 2. Methodological Approach

The biological model proposed in this review builds on the longitudinal translational research platform established at IRCCS San Raffaele Roma (Italy), as originally described by Cesario et al. [7], and subsequently developed between 2013 and 2026 through the contribution of several collaborators, bringing together complementary biological domains to better explain the heterogeneity of pulmonary rehabilitation responsiveness. It integrates the findings of these complementary investigations with current concepts in systems biology, rehabilomics, precision rehabilitation, and personalized medicine. The project involved collaborations with San Raffaele University, Rome, Italy; IRCCS San Raffaele Roma, Rome, Italy; San Raffaele Sulmona, Sulmona (L’Aquila), Italy; Sapienza University of Rome, Rome, Italy; National Institute for Infectious Diseases “Lazzaro Spallanzani” IRCCS, Rome, Italy; University of Insubria, Varese, Italy; IRCCS INRCA, Ancona, Italy; University of Naples Federico II, Naples, Italy; and the AgEA Coordinating Body (Agenzia per le Erogazioni in Agricoltura), Rome, Italy.

The present article is a narrative and conceptual synthesis rather than a systematic review. The primary evidence base consists of studies generated within the longitudinal translational research programme described above. The relevant external literature was considered to contextualize these findings within established and emerging concepts in COPD heterogeneity, precision medicine, rehabilomics, and systems biology. No formal systematic literature search or quantitative synthesis of the external literature was performed.

The programme enrolled elderly patients (>71 years old) with severe COPD (Gold stage 3–4) undergoing standardized inpatient pulmonary rehabilitation. Clinical, functional, nutritional, lifestyle, cognitive, psychological, and biological data were collected prospectively, while a dedicated biobank was established to store whole blood, serum, plasma, peripheral blood lymphocytes, DNA, and blood RNA samples (collected using PAXgene^®^ tubes from Qiagen, Milan, Italy).

Clinical and laboratory information was managed through the REDCap electronic platform, which ensured standardized data collection, quality control, and secure long-term data management [27]. The study was approved by the Ethics Committee of IRCCS San Raffaele Roma (Protocol No. 15/2013) and conducted in accordance with the Declaration of Helsinki. All participants provided written informed consent. Laboratory analyses were performed blind to clinical outcomes, and statistical analyses were conducted on pseudonymized datasets. Vital status was assessed after three and five years using municipal registry records.

As the programme evolved, successive studies explored complementary biological domains potentially associated with rehabilitation response, including oxidative stress, genomic instability, DNA damage, pharmacogenetics, cognition, behavioural resilience, nutrition, smoking-related biology, Torque Teno Virus infection, immune competence, and obesity-related inflammation. Transcriptomic, metabolomic, and lipidomic analyses are currently underway and are expected to further expand the biological characterization of the cohort.

## 3. Results

Patients with apparently similar clinical characteristics often experience markedly different outcomes after pulmonary rehabilitation, indicating that conventional clinical assessment captures only part of the biological heterogeneity of COPD. The studies reviewed here examined complementary biological domains and consistently showed that rehabilitation responsiveness reflects the interaction of genomic, immune, metabolic, behavioural, and environmental mechanisms rather than a single determinant [28,29,30,31,32,33,34,35,36,37]. The main findings are summarized below and integrated in Table 1, which provides an overview of the biological evidence generated by our longitudinal translational research programme.

### 3.1. Genomic Instability

The initial studies addressed genomic instability, a well-recognized consequence of the persistent oxidative stress associated with COPD. Elderly patients with COPD showed substantially higher levels of DNA damage than healthy older adults (19.6 ± 7.3% vs. approximately 10.5 ± 11.2% tail intensity). DNA damage increased during pulmonary rehabilitation (19.6 ± 7.3% to 21.8 ± 7.2%), despite significant improvements in exercise capacity (6MWD: 94 ± 84 to 187 ± 134 m) and pulmonary function. The increase in DNA damage was greatest in non-responders and in patients receiving long-term oxygen therapy, suggesting that genomic instability reflects biological vulnerability rather than simply disease severity [28,38].

### 3.2. Pharmacogenomics

Host genetic variability was subsequently investigated to determine whether inherited factors influenced rehabilitation responsiveness. Among the polymorphisms examined, FKBP5 was the only gene consistently associated with rehabilitation outcome. Carriers of the favourable genotype achieved greater improvements in 6MWD (152 vs. 90 m), lung function and health-related quality of life, with an approximately four-fold higher probability of successful rehabilitation. These findings suggest that pharmacogenomic variability contributes to treatment response but should be interpreted within the broader biological context of COPD [29].

### 3.3. Lifestyle and Smoking-Related Biology

Lifestyle further contributed to the biological diversity of rehabilitation outcomes. Daily vegetable consumption was independently associated with rehabilitation success (OR ≈ 3.6), with an even stronger association among patients aged ≥80 years (OR ≈ 17). Higher vegetable intake was accompanied by lower IL-6 concentrations and a more favourable inflammatory profile. Conversely, smoking identified a biological phenotype characterized by persistent DNA damage, oxidative stress, immune dysregulation and a higher prevalence of TTV viremia ≥ 4 log_10_ copies/mL, indicating that environmental exposures influence rehabilitation through biological mechanisms extending beyond smoking history alone [30,31,32].

### 3.4. Cognitive Ageing

The biological alterations identified in COPD also involved cognitive function. Patients with cognitive impairment consistently showed higher DNA damage, whereas conventional inflammatory and oxidative stress biomarkers provided limited discriminatory value. A subsequent meta-analysis confirmed the association between genomic instability and cognitive decline, supporting the concept that DNA damage reflects systemic biological ageing rather than respiratory disease alone [33].

### 3.5. Behavioural Resilience

The first evidence that behavioural response to pulmonary rehabilitation might have a biological basis came from our study on the FKBP5 rs4713916 polymorphism. Patients carrying the GA genotype showed better cognitive performance, lower anxiety and depressive symptoms, higher mental quality of life, more adaptive coping strategies, and greater functional recovery following pulmonary rehabilitation. These findings suggested that genetic factors may contribute to the individual capacity to adapt to rehabilitation.

However, genetic predisposition alone did not fully explain rehabilitation responsiveness. In a subsequent cohort of 102 elderly COPD patients, responders consistently exhibited higher Active Coping and Planning scores than non-responders, while Self-distraction was independently associated with better rehabilitation outcomes. These behavioural characteristics were also accompanied by greater functional independence, indicating that psychological adaptation contributes substantially to treatment success.

The IMPROVE pilot study further demonstrated that these behavioural traits are not fixed. A structured psychoeducational intervention increased adaptive coping, reduced maladaptive coping, and promoted the maintenance of rehabilitation benefits over time. Together, these findings indicate that behavioural resilience is both measurable and modifiable and should be considered an integral component of precision pulmonary rehabilitation programmes (Ref. [34] and manuscript in preparation).

### 3.6. Virome and Immune Ageing

The search for additional biological determinants led to the investigation of immune competence. Overall, 62.7% of patients exhibited TTV viremia ≥ 4 log_10_ copies/mL. High TTV load was associated with lower CD3+, CD4+ and CD8+ lymphocyte counts, increased regulatory T-cell frequency and reduced five-year survival. Rather than acting as a direct pathogen, TTV appears to reflect the progressive decline in immune competence accompanying advanced COPD [35,36].

### 3.7. Immune Dysfunction, Immunometabolism and Obesity

Further analyses confirmed the central role of immune regulation. Patients with poorer outcomes showed expansion of regulatory T cells, reduced lymphocyte populations and impaired cholinergic anti-inflammatory signalling, indicating that immune competence provides more meaningful prognostic information than viral burden alone [36].

The interaction between metabolism and immunity provided an additional perspective on patient heterogeneity. Approximately 26.5% of patients were obese. Compared with non-obese individuals, obese patients showed higher CD3+, CD4+ and CD8+ lymphocyte counts, lower inflammatory indices (NLR, PLR, SII and SIRI) and a lower prevalence of TTV viremia ≥ 4 log_10_ copies/mL, whereas DNA damage and five-year survival were similar between BMI groups. These findings support an immune-based interpretation of the obesity paradox, suggesting that immune–inflammatory regulation is more informative than body mass index alone for characterizing rehabilitation phenotypes [37].

### 3.8. Toward an Integrated Biological Model

The evidence reviewed here suggests that rehabilitation responsiveness in COPD cannot be explained by any single biological mechanism. Instead, it reflects the interaction of multiple processes operating at different levels of disease complexity. Genomic instability, host genetics, immune competence, persistent viral infection, immunometabolic regulation, lifestyle, behavioural resilience, cognition, and environmental exposures each provide complementary information on the biological variability underlying rehabilitation outcomes. Considered individually, none of these factors adequately captures the heterogeneity of treatment response. Their integration offers a broader biological framework for interpreting patient variability.

This integrated perspective provides the basis for moving beyond conventional clinical stratification toward a more personalized approach to pulmonary rehabilitation, in which biological and functional information jointly contribute to patient characterization and treatment planning [28,29,30,31,32,33,34,35,36,37].

## 4. Discussion

Pulmonary rehabilitation is one of the most effective non-pharmacological interventions for patients with COPD, consistently improving exercise capacity, dyspnoea and health-related quality of life [4,39,40,41,42]. Nevertheless, patients with apparently similar clinical characteristics often achieve different outcomes. This variability suggests that conventional physiological and functional assessment captures only part of the processes underlying recovery.

This observation is consistent with the current understanding of COPD. Agustí and colleagues proposed that COPD should be regarded as a heterogeneous syndrome resulting from the interaction of genetic susceptibility, environmental exposures, ageing, inflammation and impaired tissue repair rather than airflow limitation alone [43,44]. In support of this, extensive functional genomic and epidemiological studies demonstrate that polygenic lung vulnerability interacts early with environmental insults across the lifespan [45,46]. Within this framework, the molecular mechanisms of accelerated ageing and cellular senescence amplify the chronic inflammatory response, severely impairing tissue regeneration and repair capacity [47,48]. The concepts of treatable traits and, more recently, GETomics have further extended this view by emphasizing that biological mechanisms should complement clinical assessment when guiding patient management [49,50,51,52]. Along the same line, the Lancet Commission highlighted the need to move beyond a predominantly spirometric definition of COPD, emphasizing the importance of risk factors, disease trajectories, and the underlying biological mechanisms contributing to disease development and progression [10,53].

Pulmonary rehabilitation has only partly followed this evolution. Functional measures remain central to patient evaluation, whereas the biological basis of treatment response has received comparatively less attention. The studies reviewed here addressed different biological domains within the same longitudinal translational research programme, making it possible to examine molecular, clinical and behavioural findings within a common clinical setting.

Although each study addressed a specific question, the findings pointed towards a common picture. No single biomarker appeared to account for the variability in response to rehabilitation. Rather, genomic instability, host genetics, immune competence, persistent viral infection, cognition, behavioural characteristics, lifestyle and obesity were associated with different aspects of patient heterogeneity. Importantly, these findings are mainly based on observational studies and therefore do not allow causal relationships to be established. These factors should be considered as possible contributors to differences in rehabilitation response rather than as established determinants or validated predictors of outcome. It is also important to recognize that responsiveness to pulmonary rehabilitation is not a single or uniform outcome. Patients may improve to different degrees in exercise capacity, symptoms, health-related quality of life, functional status, or other clinical domains, and improvement in one domain does not necessarily imply a similar response in another. The different outcomes used across the studies in this programme should therefore be considered when interpreting the apparent convergence of the findings.

This interpretation is also in keeping with the emerging literature on biomarkers and exercise-based pulmonary rehabilitation. The biological characteristics identified in our studies may provide complementary information for understanding differences between patients, but their value for predicting individual responses or guiding treatment decisions remains to be established.

A recent narrative review by Candia et al. [26] summarized evidence on inflammatory and oxidative stress markers, DNA damage, microbiota and metabolic signatures as potential biomarkers of the biological response to rehabilitation. These studies suggest that biomarkers may provide information that is not fully captured by conventional clinical and functional measures and may have a future role in patient phenotyping and in monitoring changes during rehabilitation. However, the evidence remains heterogeneous, and the clinical meaning of individual biomarker changes is not yet sufficiently established. Differences in patient characteristics, rehabilitation protocols, timing of sampling and laboratory methods make it difficult to compare findings across studies and to identify biomarkers that can be reliably used in clinical practice [26]. Thus, biomarker-based approaches should currently be considered complementary to, rather than a replacement for, established clinical and functional assessment.

Genomic instability was among the first biological alterations identified in this programme. DNA damage remained elevated during rehabilitation despite improvements in exercise capacity, pulmonary function and health-related quality of life [28]. Similar observations have been reported in elderly individuals exposed to chronic oxidative stress, where biological adaptation may follow a different time course from functional recovery [38,54] and in other cohorts of COPD patients undergoing physical exercise [55]. FKBP5 polymorphisms identified patients with greater rehabilitation benefit, whereas the remaining candidate genes showed limited additional value [29]. These findings suggest that host genetic variability contributes to rehabilitation outcome but does not explain it on its own.

Lifestyle and behavioural factors added another perspective. Regular vegetable consumption was associated with lower systemic inflammation and better rehabilitation outcomes, whereas smoking was associated with persistent oxidative stress, DNA damage and immune alterations [30,31,32]. Our observations are consistent with other studies investigating the protective impact of vegetable consumption on COPD development [56,57] as well as the effect of smoking on inflammation and immune response [58]. Cognitive impairment, which in COPD can be manifested by functional disability, poor psychological well-being, frequent memory loss and faster cognitive decline over time [59,60,61], in our cohort was associated with increased genomic damage, while patients with better outcomes consistently reported more adaptive coping strategies [33,34]. Considered individually, these observations provide only part of the explanation; viewed together, they indicate that behavioural and biological factors contribute jointly to rehabilitation outcome.

The studies on immune competence reached similar conclusions. Higher Torque Teno Virus (TTV) viraemia was associated with increased genomic instability, impaired immune status and reduced long-term survival [35,36]. These findings are consistent with the interpretation of TTV as a marker of immune competence rather than a direct pathogenic factor [62,63]. Likewise, obese patients showed lower systemic inflammation, lower TTV viraemia and better preservation of immune function despite similar survival [37]. These observations support the role of immune regulation as one of the mechanisms contributing to patient variability.

This interpretation is consistent with recent developments in precision medicine. The Lancet Commission proposed a biological framework integrating disease mechanisms and risk factors [10]. Recent reviews further support this view. Finney et al. proposed that precision treatment should target the appropriate biological pathway in the appropriate patient, whereas Xie et al. highlighted the integration of phenotypes, endotypes, biomarkers and multi-omics technologies to improve patient stratification in COPD [64,65]. Although these models were developed mainly for disease classification and targeted therapies, the same principles may also be relevant to pulmonary rehabilitation.

In this context, the concept of rehabilomics is particularly relevant. Candia et al. [26] highlighted how the integration of molecular information with rehabilitation outcomes may help describe the biological processes underlying recovery and, in the future, support more individualized rehabilitation strategies. Their review also emphasized that this field is still developing: promising findings have been reported for oxidative stress, DNA damage, microbiota and metabolic markers, but larger studies, standardized methods and independent validation are needed before these approaches can be translated into routine practice [26]. Our findings fit within this emerging framework, while also supporting a broader view in which biological markers are considered together with clinical, functional, behavioural and lifestyle characteristics rather than as isolated predictors of response [26].

Multi-omics approaches represent a further development of this framework, but they should currently be considered as a future extension of the research programme rather than as part of the empirical evidence presented in this review. The published findings summarized here derive from the biological, clinical, functional, behavioural and lifestyle domains described above, whereas transcriptomic, metabolomic and lipidomic analyses are currently ongoing. These approaches may provide additional information on the molecular pathways associated with different patterns of rehabilitation responsiveness and may help to refine the biological framework proposed here.

Metabolomics is particularly promising in this regard. Recent evidence indicates that metabolic profiling may contribute to the characterization of COPD phenotypes and severity and may identify pathways related to inflammation, oxidative stress, energy metabolism and other disease mechanisms [66,67,68]. Metabolomic approaches have also been investigated for biomarker discovery and prognosis, supporting their potential relevance to precision medicine. However, several methodological issues remain. Results may be influenced by the biological matrix used, sample handling and stability, intra- and inter-individual variability, dilution effects, analytical platform and data-processing procedures. Moreover, no single analytical platform can comprehensively capture the entire metabolome, and differences in sampling and analytical methods may limit comparisons between studies [68,69]. The current evidence therefore supports the potential value of metabolomics, but also indicates that its clinical application requires careful standardization and independent validation.

For these reasons, transcriptomics, metabolomics and lipidomics should currently be regarded as promising tools for the future refinement of patient phenotyping and response assessment rather than as validated methods for clinical stratification. Their integration with the biological and clinical information already available may eventually provide a more comprehensive view of the pathways associated with rehabilitation response. However, this hypothesis will need to be tested through longitudinal studies and independent cohorts before these approaches can be translated into clinical practice.

Overall, the available evidence suggests that rehabilitation outcome cannot be explained by a single biological factor. Different biological characteristics may contribute to the variability observed among patients, but the available studies do not establish causal relationships or provide sufficient evidence to support validated prediction of rehabilitation response. Rather, the findings support a multidimensional framework in which biological, clinical, functional, behavioural and lifestyle characteristics are considered together as complementary sources of information.

An important feature of this work is the continuity of the research programme. Clinical, functional, molecular and psychological data were collected over more than ten years using standardized rehabilitation protocols and harmonized laboratory procedures. This allowed findings from different studies to be interpreted within the same research framework and reduced some of the methodological variability that often limits comparisons across independent investigations. At the same time, this continuity should not be interpreted as equivalent to external validation. The evidence comes predominantly from a single longitudinal translational programme involving a relatively selected population of older patients with severe COPD. This limits the generalizability of the findings to younger patients, patients with less severe disease and other rehabilitation settings. Moreover, the proposed biological framework has not yet been independently validated in multicenter cohorts. Further studies using standardized protocols and broader COPD populations will therefore be needed to establish whether the biological domains identified here can meaningfully improve phenotyping, monitoring and prediction of response to pulmonary rehabilitation.

Several limitations should be taken into account when interpreting these findings. The evidence reviewed comes mainly from a longitudinal research programme conducted in a single clinical setting and involving elderly patients with severe COPD undergoing standardized inpatient pulmonary rehabilitation. This specific patient population may limit the generalizability of the findings to younger patients, those with less severe disease, and individuals undergoing pulmonary rehabilitation in other clinical settings. In addition, several studies originated from the same research programme and involved similar, and potentially partially overlapping, patient populations. Therefore, the consistency of findings across studies should not be interpreted as independent replication, but rather as complementary observations obtained within a relatively consistent clinical and methodological context.

Another important consideration is that responsiveness to pulmonary rehabilitation is not a single or uniform outcome. The studies included in this programme used different measures to characterize response, including exercise capacity, pulmonary function, symptoms, health-related quality of life, cognitive and psychological outcomes, and longer-term clinical outcomes. Patients may therefore improve in some domains but not others, and the use of different outcomes needs to be considered when interpreting the apparent convergence of the findings.

The single-center design is an important limitation of this work. Although the longitudinal approach and the use of standardized rehabilitation protocols allowed us to assess different biological and clinical domains within a consistent setting, the findings cannot be assumed to be generalizable to other populations or rehabilitation settings. In addition, several studies were conducted within the same longitudinal research programme and included similar, and potentially partially overlapping, patient populations. Thus, the consistency observed across studies should be interpreted as complementary evidence rather than as independent replication.

Another consideration is the multidimensional nature of the response to pulmonary rehabilitation. The studies included in this programme used different outcomes to characterize response, including exercise capacity, pulmonary function, symptoms, health-related quality of life, cognitive and psychological measures, and longer-term clinical outcomes. Patients may therefore improve in some domains but not others, and these differences should be taken into account when interpreting the overall pattern of findings.

Taken together, these considerations mean that the biological model proposed here should currently be viewed as a hypothesis-generating framework rather than as a validated model for predicting rehabilitation response or guiding patient stratification. Independent prospective and multicenter studies involving broader COPD populations and different rehabilitation settings will be needed to determine whether the biological and clinical domains identified in this programme can reliably contribute to patient phenotyping and response assessment and, ultimately, support more individualized pulmonary rehabilitation.

## 5. Conclusions

Clinical improvement after pulmonary rehabilitation reflects more than changes in functional performance alone. The available evidence suggests that rehabilitation outcomes are influenced by multiple biological processes that complement conventional clinical assessment. Integrating molecular, immune, behavioural and clinical information may support a more individualized approach to pulmonary rehabilitation.

Ongoing transcriptomic, metabolomic and lipidomic studies will further characterize the biological basis of rehabilitation outcomes. Their integration with clinical and functional data will refine the proposed biological model. Validation in independent multicentre cohorts will be an essential next step before clinical application.

## Figures and Tables

**Table 1:** Biological domains investigated within the longitudinal translational research programme and their contribution to precision pulmonary rehabilitation.

Biological Domain	Major Biological Findings	Evidence Status	Contribution to Precision Pulmonary Rehabilitation	Reference(s)
Genomic Instability	In 89 elderly patients with COPD undergoing a 3-week pulmonary rehabilitation programme, baseline DNA damage was markedly elevated compared with healthy older adults. Pulmonary rehabilitation significantly improved 6MWD, FEV1, dyspnoea, MRC and SGRQ despite a significant increase in DNA damage. The increase was greater in non-responders and in patients receiving long-term oxygen therapy. DNA damage correlated positively with baseline pCO2 and inversely with IL-6.	Published	Genomic instability may represent an early marker of biological vulnerability. The dissociation between functional improvement and persistent or increased DNA damage highlights the potential value of integrating molecular markers with conventional functional outcomes.	[28]
Pharmacogenomics	In 71 elderly patients with COPD, FKBP5 rs4713916 was the only polymorphism associated with rehabilitation responsiveness. GA carriers showed greater improvements in 6MWD, FEV1 and SGRQ and had an approximately four-fold higher probability of successful rehabilitation.	Published	FKBP5 may represent a potential genetic marker of differential rehabilitation response and may contribute to future individualized rehabilitation and treatment strategies.	[29]
Lifestyle and Smoking-Related Biology	In 105 elderly patients with COPD, daily vegetable consumption was more frequent among responders and was independently associated with rehabilitation success, particularly in patients aged ≥80 years. Higher vegetable intake was associated with a more favourable inflammatory profile. In 102 patients, current smoking was associated with greater oxidative DNA damage, increased 8-OHdG, lower bilirubin, immune remodelling and a higher prevalence of TTV ≥ 4 log10 copies/mL.	Published	Lifestyle factors, particularly diet, may influence systemic inflammation and rehabilitation responsiveness. Smoking-related biological alterations support the assessment of biological vulnerability beyond smoking status alone.	[30,31,32]
Cognitive Ageing	In 96 elderly patients with COPD, cognitive impairment was associated with significantly higher DNA damage. Genomic instability independently predicted cognitive impairment and temporal orientation deficits. Meta-analysis confirmed an association between DNA damage and cognitive decline.	Published	Combining biological and cognitive assessment may help identify vulnerable patients and support more tailored rehabilitation strategies.	[33]
Behavioural Resilience	The FKBP5 rs4713916 GA genotype was associated with better cognitive performance, lower anxiety and depression, higher mental quality of life, more adaptive coping strategies and greater functional recovery. In a subsequent cohort of 102 elderly patients, Active Coping and Planning were the strongest behavioural predictors of rehabilitation responsiveness, while Self-Distraction was independently associated with better outcomes. These findings were subsequently translated into the IMPROVE pilot trial, in which a structured psychoeducational intervention increased adaptive coping and improved maintenance of rehabilitation benefits.	Published/manuscript in preparation	Behavioural resilience represents a potentially measurable and modifiable determinant of rehabilitation responsiveness. Behavioural phenotyping combined with targeted psychoeducational interventions may support individualized rehabilitation and long-term maintenance of benefits.	[34]; manuscript in preparation
Virome and Immune Ageing	In 102 elderly patients with COPD, 62.7% had TTV ≥ 4 log10 copies/mL. High TTV load was associated with lower CD3+, CD4+ and CD8+ lymphocytes, increased Treg frequency, reduced α7 nicotinic acetylcholine receptor expression, VZV coinfection and shorter 5-year survival. Treg frequency was the strongest independent predictor of mortality.	Published	TTV may provide a functional marker of immune competence and immunosenescence. Combining TTV load with immune phenotyping may improve biological characterization and prognostic stratification.	[35]
Immune Dysfunction, Immunometabolism and Obesity	In 70 elderly patients with COPD, obesity was associated with higher lymphocyte, CD3+, CD4+ and CD8+ T-cell counts and lower NLR, PLR, SII and SIRI. TTV ≥ 4 log10 copies/mL was less frequent in obese patients, whereas oxidative stress, DNA damage and 5-year survival did not differ between BMI groups.	Published	Obesity appears to modify the biological phenotype of COPD, with preservation of immune competence and lower systemic inflammation, supporting an immune-based interpretation of the obesity paradox.	[36,37]
Transcriptomics	Transcriptomic analyses are being undertaken to investigate molecular pathways potentially associated with rehabilitation responsiveness and to complement the biological information generated by the published studies.	Ongoing	May provide additional information on molecular pathways underlying different patterns of rehabilitation response and contribute to the future refinement of biological phenotyping.	Ongoing study
Metabolomics	Metabolomic analyses are currently ongoing within the research programme. Their aim is to identify metabolic signatures and pathways potentially associated with rehabilitation responsiveness.	Ongoing	May provide complementary information on metabolic pathways involved in rehabilitation response and may contribute to future patient phenotyping and response monitoring.	Ongoing study
Lipidomics	Lipidomic analyses are currently ongoing and are intended to characterize lipid-related pathways potentially associated with COPD heterogeneity and rehabilitation responsiveness.	Ongoing	May complement other biological domains by providing information on lipid metabolism and its potential relationship with rehabilitation response.	Ongoing study
Integrated Biological Model	The proposed model integrates the published findings on genomic instability, pharmacogenomics, lifestyle and smoking-related biology, cognitive ageing, behavioural resilience, virome and immune ageing, and immune dysfunction/immunometabolism associated with obesity. Transcriptomic, metabolomic and lipidomic analyses represent the next phase of the research programme and have not yet been incorporated as empirical evidence supporting the model.	Conceptual framework; multi-omics component ongoing	Provides a multidimensional framework for biological phenotyping and individualized pulmonary rehabilitation, while generating hypotheses for future validation.	Present review

Note: Evidence status refers to the stage of development within the longitudinal translational research programme. “Published” indicates findings already reported in peer-reviewed studies; “Ongoing” refers to analyses currently in progress; and “Conceptual framework” indicates hypotheses or future directions that have not yet been empirically validated. Transcriptomic, metabolomic and lipidomic analyses are therefore presented as ongoing components of the research programme and should not be interpreted as evidence already supporting the proposed integrated model.

## Data Availability

This manuscript is a narrative review. No new datasets were generated or analyzed. The evidence supporting the conclusions is derived from the published literature comprehensively cited in the reference list.

## Abbreviations

The following abbreviations are used in this manuscript:

Abbreviation	Definition
6MWD	Six-Minute Walk Distance
BMI	Body Mass Index
CAT	COPD Assessment Test
COPD	Chronic Obstructive Pulmonary Disease
DNA	Deoxyribonucleic Acid
FEV1	Forced Expiratory Volume in One Second
FKBP5	FK506 Binding Protein 5
GOLD	Global Initiative for Chronic Obstructive Lung Disease
ICF	International Classification of Functioning, Disability and Health
IL-6	Interleukin-6
LMR	Lymphocyte-to-Monocyte Ratio
MRC	Medical Research Council Dyspnoea Scale
NIH	National Institutes of Health
NLR	Neutrophil-to-Lymphocyte Ratio
OR	Odds Ratio
PLR	Platelet-to-Lymphocyte Ratio
REDCap	Research Electronic Data Capture
SGRQ	St George’s Respiratory Questionnaire
SII	Systemic Immune–Inflammation Index
SIRI	Systemic Inflammation Response Index
Treg	Regulatory T Cell
TTV	Torque Teno Virus
VZV	Varicella-Zoster Virus
WHO	World Health Organization
α7nAChR	Alpha-7 Nicotinic Acetylcholine Receptor
